# Reconceptualizing Pediatric Strabismus as a Condition Rooted in Sensory Processing Disorder: A Novel Case-Based Hypothesis

**DOI:** 10.3390/children12070904

**Published:** 2025-07-09

**Authors:** Mirjana Bjeloš, Ana Ćurić, Mladen Bušić, Katja Rončević, Adrian Elabjer

**Affiliations:** 1 University Eye Department, Reference Center of the Ministry of Health of the Republic of Croatia for Pediatric Ophthalmology and Strabismus, Reference Center of the Ministry of Health of the Republic of Croatia for Inherited Retinal Dystrophies, Reference Center of the Ministry of Health of the Republic of Croatia for Standardized Echography in Ophthalmology, University Hospital “Sveti Duh”, Sveti Duh 64, 10000 Zagreb, Croatia; mbjelos@kbsd.hr (M.B.); mbusic@kbsd.hr (M.B.); 2 Faculty of Medicine, Josip Juraj Strossmayer University of Osijek, 31000 Osijek, Croatia; 3 Faculty of Dental Medicine and Health Osijek, Josip Juraj Strossmayer University of Osijek, 31000 Osijek, Croatia; 4 School of Medicine, Catholic University of Croatia, 10000 Zagreb, Croatia; kroncevic@unicath.hr; 5 School of Medicine, University of Zagreb, 10000 Zagreb, Croatia; aelabjer@student.mef.hr

**Keywords:** strabismus, sensation disorders, blinking, tics, hyperactivity disorders, early diagnosis, treatment, autism

## Abstract

**Background/Objectives:** A direct link between sensory processing disorder (SPD) and strabismus has not been systematically investigated, though prior studies suggest sensory modulation may influence visual behaviors. Traditional approaches view strabismus through a binary lens—either normal or pathological motor deviation. This report presents a proof-of-concept case suggesting strabismus may represent a neurobehavioral manifestation of sensory processing imbalance, rooted within the broader framework of SPD. **Methods:** We report a pediatric case marked by episodic monocular eye closure triggered by environmental stimuli, without identifiable ophthalmologic or neurologic pathology. The child’s symptoms were most consistent with sensory over-responsivity (SOR), a subtype of SPD, manifesting as stimulus-bound monocular eye closure and secondary self-regulatory behaviors. **Results:** We propose the *Fusion Dysregulation Hypothesis,* suggesting that exotropia and esotropia represent opposing outcomes along a continuum of sensory connectivity: exotropia arising from neural underwiring (hyporesponsivity and fusion instability), and esotropia from overwiring (hyperresponsivity and excessive fusion drive). Our case, marked by sensory hyperresponsivity, showed frequent monocular eye closure that briefly disrupted but did not impair fusion. This suggests an “overwired” binocular system maintaining single vision despite sensory triggers. In early-onset esotropia, such overconnectivity may become maladaptive, leading to sustained convergence. Conversely, autism spectrum disorder, typically associated with hypoconnectivity, may predispose to exotropia through reduced fusion maintenance. **Conclusions:** These findings highlight the need for interdisciplinary evaluation. We advocate for structured sensory profiling in children presenting with strabismus and, conversely, for ophthalmologic assessment in those diagnosed with SPD. While our findings remain preliminary, they support a bidirectional screening approach and suggest that sensory modulation may play a previously under-recognized role in the spectrum of pediatric strabismus presentations.

## 1. Introduction

Strabismus is among the most frequently encountered conditions in pediatric ophthalmology, affecting an estimated 2–5% of children globally [1,2,3,4]. Traditionally conceptualized as a disorder of ocular motor alignment, strabismus is clinically classified into forms such as esotropia (inward deviation) and exotropia (outward deviation) [5]. However, converging evidence from developmental neuroscience and neurodevelopmental disorders—including autism spectrum disorder (ASD) and sensory processing disorder (SPD)—increasingly suggests that the etiology of certain visual behaviors may arise from atypical sensory integration rather than from purely structural or anatomical misalignments of the extraocular musculature [6].

SPD is characterized by the brain’s impaired capacity to modulate, discriminate, or integrate sensory input across one or more modalities, resulting in maladaptive responses to everyday sensory stimuli [7]. Initially conceptualized by occupational therapist A. Jean Ayres in 1972 as “sensory integration dysfunction” [8], and subsequently termed SPD by Miller in 2006 [9], the condition is not formally recognized in the Diagnostic and Statistical Manual of Mental Disorders, Fifth Edition (DSM-5) [10] or the International Classification of Diseases (ICD-11) [11]. However, controversy persists regarding SPD’s status as an independent diagnosis; notably, an American Academy of Pediatrics policy statement [12] advised against using SPD as a stand-alone diagnosis despite its widespread clinical use [12,13]. Nonetheless, many clinicians and therapists continue to recognize and treat SPD as a distinct entity in practice [12,14]. Epidemiological studies estimate that features associated with SPD—such as extreme sensory sensitivities or under-responsiveness—may affect 5–16% of school-aged children, making it as common as or exceeding the prevalence of more widely recognized neurodevelopmental conditions [15]. Importantly, atypical sensory processing has been documented in up to 90% of individuals with ASD and in approximately 50% of children with attention-deficit/hyperactivity disorder (ADHD) [7]. SPD, however, may also occur in the absence of ASD or ADHD, leading to continued debate regarding whether SPD represents an independent clinical entity or a manifestation of broader neurodevelopmental syndromes [16].

ASD is a complex neurodevelopmental condition characterized by persistent deficits in social communication and social interaction across multiple contexts, along with restricted, repetitive patterns of behavior, interests, or activities [10]. Individuals with ASD often exhibit differences in sensory processing, including hyper- or hypo-reactivity to sensory input or unusual interest in sensory aspects of the environment [10]. These sensory features are now recognized as core diagnostic elements of ASD and may profoundly influence visual, auditory, and tactile experiences [10]. Such sensory modulation difficulties frequently overlap with those observed in SPD, although ASD is defined by a broader constellation of social-communicative and behavioral features.

Visual processing represents a sensory modality commonly impacted in both SPD and ASD [7]. In particular, children with sensory over-responsivity may experience visual hypersensitivity, which manifests as heightened distress or behavioral avoidance in environments with intense or complex visual stimuli [7]. Adaptive responses to such hypersensitivity may include squinting, shielding, or closing one eye, or seeking darker environments [17]. These behaviors may serve as compensatory mechanisms to attenuate overwhelming sensory input and can be conceptualized as a “flight” response to excessive visual stimulation [7]. Of particular note is the phenomenon of monocular eye closure—a behavior that effectively halves the incoming visual information to the brain [18]. In clinical ophthalmology, a history of a child closing one eye in response to bright light typically raises concern for intermittent exotropia, as such behavior may reflect the disruption of binocular fusion under high-intensity lighting conditions [17].

We report a pediatric case marked by episodic monocular eye closure triggered by environmental stimuli, in the absence of identifiable ophthalmologic or neurologic pathology. The most consistent explanation was SPD, specifically sensory over-responsivity (SOR) [19], manifesting as stimulus-bound monocular eye closure and secondary self-regulatory behaviors. Based on this case, we propose a novel *Fusion Dysregulation Hypothesis*, suggesting that strabismus may represent a neurobehavioral manifestation of sensory processing imbalance, with exotropia and esotropia representing opposing outcomes along a continuum of sensory connectivity. Notably, subtle visual-oculomotor anomalies have been reported even in children with isolated SPD who have otherwise normal eye exams [20], supporting the plausibility of sensory-driven visual behaviors in the absence of structural pathology. Supporting our hypothesis, experimentally altering early visual experience in infant primates can induce strabismus through sensory-driven mechanisms [21]. Furthermore, children with strabismus (with or without amblyopia) have shown abnormal functional connectivity in brain regions subserving visual and sensory integration [6].

## 2. Case Report

### 2.1. Patient Description

**Figure 1:**
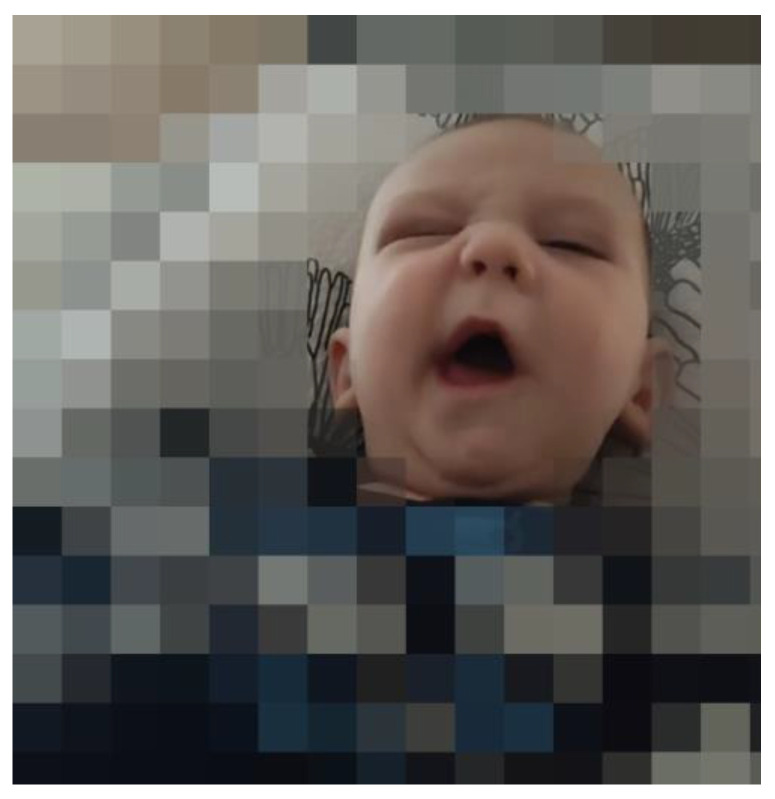
The patient presented unilateral blinking, facial grimacing, and contraction of the perioral muscles at 2 months of age.

A male infant, born in August 2022, with an unremarkable perinatal history and typical early developmental milestones, initially presented at 8 months of age with intermittent blinking of the right eye. These brief episodes, lasting one to two seconds, occurred primarily in response to bright sunlight. On retrospective video recordings review, earlier subtle signs had been present: as early as 2 months of age, the infant often kept his right eye closed during breastfeeding—particularly when nursing on the left side. Subsequently, he exhibited a marked preference for the left breast, persistently rejecting the right. These behaviors were intermittently accompanied by unilateral blinking, facial grimacing, and contraction of the perioral muscles, suggesting early sensorimotor responses (Figure 1). These manifestations were more prominent on the right side, though similar features were occasionally observed on the left.

Importantly, the evolution of symptoms appeared to parallel the child’s neurodevelopmental progression (Table 1).

As he gained greater visual, sensory, and motor awareness between 8 and 10 months of age, monocular eyelid closures became more pronounced and prolonged. Environmental stimuli such as sunlight, camera flashes, air currents (e.g., from an inhaler), and wind increasingly provoked eye squeezing, primarily on the right side. During exposure to winter snow, the infant tightly closed his right eye, rubbed his forehead, and became agitated, though without overt crying. Once indoors and away from the stimulus, these behaviors abated rapidly.

**Figure 2:**
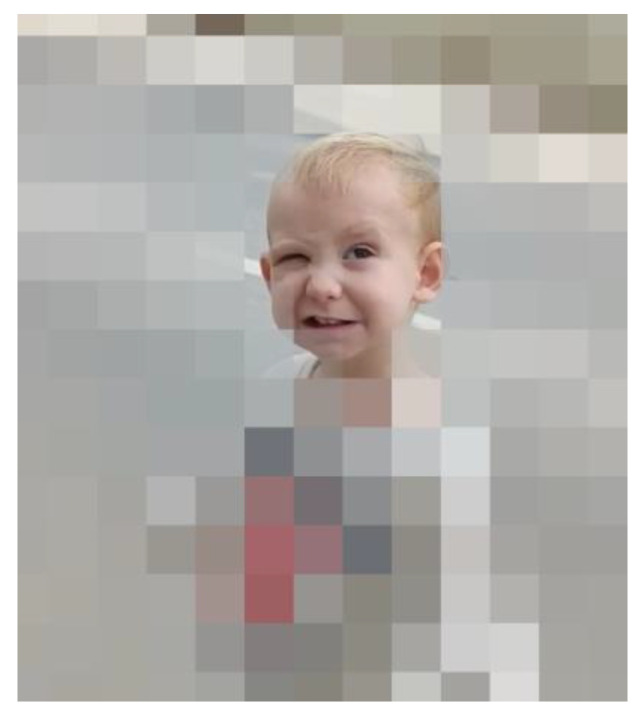
Eye closure caused by wind at the age of 2 years.

By 10 months of age, during spoon-feeding, he partially closed one eye and displayed chewing-associated facial movements. Between 1 and 2 years of age during teething flares, the child’s threshold for sensory triggers decreased, with even moderate light or air movement prompting eye closure and facial rubbing (Figure 2). Somatosensory triggers—including vibration from sitting on a digger—elicited similar responses, sometimes affecting either eye.

**Figure 3:**
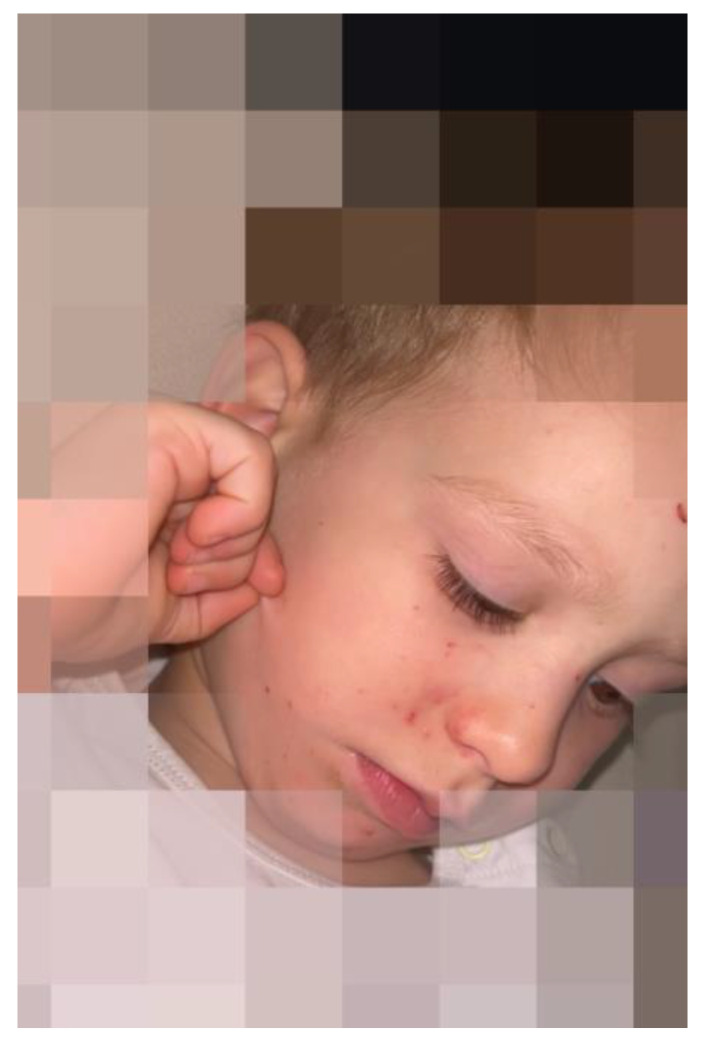
Face pinching while watching the screen by 3 years of age.

At these times, he demonstrated self-soothing behaviors such as pinching the skin around his right eye or pulling his hair, particularly when multiple discomforts co-occurred (e.g., teething pain and visual stimulation). These actions resembled motor tics or dystonic spasms but were interpreted as adaptive strategies to manage sensory overload. By age 3, with the completion of primary tooth eruption, although visual hypersensitivity persisted, general irritability decreased. New compensatory habits developed: while watching television, he frequently rested the right side of his face on his fist, occluding the right eye—particularly during fast-paced or brightly colored sequences. On handheld screens, viewing time was self-limited to 1–2 min before he looked away and rubbed his eyes or forehead, pinched his face and pull hair, often appearing overstimulated (Figure 3).

He also began actively avoiding discomfort: he consistently requested dark UV400 sunglasses before going outdoors on bright days, which markedly reduced the need for squinting or rubbing. Additionally, he instinctively shielded his face with his hands in windy environments. At no point did the child lose awareness or responsiveness, excluding absence seizures. He never described light or wind as painful, but instead used phrases such as “too bright” or simply refused to open the affected eye until the stimulus had passed.

### 2.2. Clinical Evaluations

Across multiple evaluations, ophthalmologic examinations remained normal. Visual tracking and fixation were age-appropriate. No manifest or intermittent strabismus was observed. Anterior segment examination revealed no corneal or lenticular opacities, and fundoscopy was unremarkable. Neurological evaluations were similarly unrevealing. Brain MRI demonstrated no abnormalities, including no lesions or vascular compression involving the facial nerve. Electroencephalogram (EEG) during wakefulness and sleep showed no epileptiform activity. Electromyography (EMG) of the facial musculature revealed no signs of myopathy or neuropathy; there was no evidence of hemifacial spasm or aberrant facial nerve regeneration.

### 2.3. Differential Diagnosis

Benign Essential Blepharospasm (BEB), although exceedingly rare in pediatric populations [22], was considered due to the recurrent eye closure. However, the patient’s episodes lacked the hallmark features of BEB, including involuntary, tonic orbicularis oculi spasms. Instead, eye closure appeared volitional, context-dependent, and behaviorally modulated, inconsistent with the involuntary dystonic contractions characteristic of BEB.

Inverted Marcus Gunn jaw-winking synkinetic phenomenon (Marin–Amat syndrome) was suspected because the child often demonstrated a sustained blink during feeding or sucking [23]. However, detailed observation showed no consistent linkage between jaw movement and eyelid position; chewing did not invariably elicit eye closure, and conversely, eye closure occurred often without any jaw motion. These findings effectively ruled out a trigemino-facial synkinesis.

Trigeminal sensory hyperexcitability, photophobia, and migraine variants were other considerations, especially during the period of primary toothing. This seemed less likely since he never showed signs of actual pain, and neurological exams were normal.

The patient’s blinking and facial grimacing initially resembled simple motor tics. However, tic behaviors are typically suppressible, episodic, and not consistently tied to external stimuli [24]. In contrast, this patient’s eye closures were reliably stimulus-bound. Moreover, there were no associated vocal tics, spontaneous stereotypies, or waxing–waning patterns suggestive of Tourette syndrome [25]. Neurologic examination and EEG were normal, providing no evidence of a tic disorder or underlying seizure activity.

Halpern Syndrome (Monocular Vestibular Vertigo) involves episodic vertigo with a perceptual tilt, and reflexive monocular eye closure contralateral to a vestibular lesion [26]. However, the patient exhibited no signs of vertigo, postural instability, or head position sensitivity. Gait, balance, and vestibular reflexes were age-appropriate, effectively excluding a vestibular etiology.

Ochoa (Urofacial) Syndrome is a rare genetic disorder characterized by a paradoxical “inverted” facial expression during smiling and severe lower urinary tract dysfunction [27]. It was briefly considered in light of early atypical facial movements. However, the child’s facial expressions were reactive to sensory stimuli rather than affective or social cues, and there were no genitourinary abnormalities. Thus, Ochoa Syndrome was excluded.

After thorough evaluation and exclusion of structural, neurologic, and synkinetic disorders, the most consistent explanation was SPD, specifically SOR, manifesting with stimulus-bound monocular eye closure and secondary self-regulatory behaviors. The family was counselled regarding the diagnosis, and the patient was referred to a pediatric occupational therapist for formal sensory integration assessment and intervention planning.

## 3. Discussion

We describe a pediatric patient with episodic monocular eye closure and facial discomfort triggered predominantly by visual stimuli (bright light, high contrast visual environments) and to a lesser extent by tactile (e.g., wind, snowflakes on the face) and vestibular-proprioceptive stimuli (e.g., vibration), consistent with SOR multiple modalities [19]. The absence of any structural or neuro-ophthalmic abnormality, combined with the clear sensory precipitating factors, led us to attribute the child’s symptoms to SPD—specifically, an over-reactivity to visual input resulting in compensatory behaviors. To our knowledge, this is the first reported case highlighting isolated visual-domain SPD presenting with eye closure behavior mimicking intermittent exotropia and ocular dystonia. This case prompts a re-examination of how sensory modulation difficulties can masquerade as or coexist with traditional ophthalmic diagnoses.

SPD refers to a condition in which the brain does not properly organize and respond to stimuli from one or more sensory modalities [7]. In 2007, Miller et al. proposed a nosology dividing SPD into subtypes: Sensory Modulation Disorder (problems turning sensory input up or down, which includes over-responsivity, under-responsivity and sensory seeking/craving), Sensory Discrimination Disorder (difficulty interpreting the qualities of sensory stimuli), and Sensory-Based Motor Disorder (issues with postural or volitional movement due to poor processing) [19]. Our patient’s presentation is most consistent with a sensory modulation problem—specifically SOR. In SOR, stimuli that are tolerable to most people evoke exaggerated responses of discomfort, avoidance, or distress [7]. The child exhibited classic features of over-responsivity in the visual system: he found normal levels of light or visual movement aversive and responded with a “fight or flight” behavior (in his case, a *flight* response—closing the eye or escaping the stimulus, sometimes accompanied by a *fight* component—rubbing or pinching himself due to frustration).

It is notable that the first signs appeared in infancy, which aligns with SPD being a neurodevelopmental issue rather than an acquired illness [16]. From birth, infants differ in their sensory thresholds; some are far more sensitive to lights, sounds, and touch [5]. In retrospect, our patient’s early refusal to open his right eye during feeding might have been an instinctual way to reduce the overall sensory load while engaging in the complex act of nursing, which itself involves multiple sensory inputs like taste, smell, touch, and vestibular sensations. As he grew, his sensory world became more complex (e.g., crawling outdoors, encountering wind, snow and bright sunshine, exposure to screen media), and his symptoms evolved from simple blinking to more elaborate protective maneuvers (covering one eye, seeking dark glasses) and self-soothing behaviors (rubbing forehead, pulling hair). This progression illustrates the development of coping strategies that, while unusual in appearance, were purposeful adaptations to overwhelming sensory input (Table 1). The squeezing of the eyes, facial grimacing, and hair pulling in this case could easily be mistaken for a tic disorder or a form of focal seizure. However, the differentiating factor was the consistency of environmental triggers—the child did not exhibit these behaviors randomly; they were predictably provoked by specific sensory contexts, and when the stimulus was removed (e.g., coming indoors from bright sun, or wearing sunglasses), the behaviors ceased immediately.

Our patient’s symptom profile aligns with the known SPD phenotype of visual over-responsivity described in the literature [14,16,19]. In other words, he represents an SPD case primarily affecting the visual domain, without co-occurring global developmental disorders. Such cases underscore that severe sensory modulation issues can exist as an isolated condition in an otherwise typically developing child—a point of ongoing debate in the field [16]. By definition, the child in this report does not meet criteria for autism or any global developmental disorder—he has normal social-communication skills for his age and no behavioral diagnoses. Yet he clearly struggles with sensory regulation. Historically, SPD has often been discussed in the context of autism or ADHD, given the high prevalence of sensory symptoms in those populations, leading some clinicians to assume that severe sensory issues only occur as part of those diagnoses [7]. Recent research supports that SPD symptoms can exist on their own as a primary disorder of sensory brain circuits. Neuroimaging work by Owen et al. demonstrated that children with SPD who had no autism or ADHD showed distinctive reductions in white matter integrity in posterior brain regions involved in multisensory integration, suggesting a unique neural signature of SPD [15]. This adds objective validity to SPD as a standalone condition—one that may be overlooked if we focus only on classic diagnoses. In our patient, recognizing SPD was crucial to guiding therapy (toward sensory integration strategies rather than unnecessary neurological interventions).

From a visual system perspective, this case raises intriguing questions about the interface between sensory processing and ophthalmology. Monocular eye closure in children is classically associated with intermittent exotropia, where bright light can precipitate an outward deviation of one eye along with the child closing that eye [17]. The mechanism in exotropia is thought to be that strong light “dazzles” the retina and temporarily overwhelms fusion, causing the brain to suppress one eye’s input [18]. Our patient, interestingly, exhibited the behavior of closing one eye in bright light without actually having any strabismus. This suggests that sensory overload alone—in the absence of a motor misalignment issue—can drive a child to shut one eye. It is reasonable to hypothesize that in intermittent exotropia patients, the fundamental issue might not only be neuromuscular misalignment, but also an element of sensory intolerance: i.e., some children with intermittent exotropia could have lower tolerance for intense visual stimulation and thus more readily break fusion and close an eye under bright conditions. In other words, sensory processing dysfunction may contribute to the clinical expression of intermittent exotropia. We found no prior publications explicitly proposing this link. However, considering that both intermittent exotropia and SPD can involve disruptions in binocular visual processing and attention, it is plausible that they share common neural underpinnings. For example, children with convergence insufficiency or intermittent exotropia have been noted to have higher rates of attention and learning issues [13,28], and conversely, children with neurodevelopmental disorders often have subtle ocular motor anomalies [14].

Another aspect highlighted by this case is the repertoire of self-regulation behaviors the child used. When faced with an unbearable sensory input, the child not only closed his eyes but also engaged in actions like pressing on his eye area, rubbing and pinching his face, or pulling his hair. However, while other signs of self-regulation gradually emerged, the act of closing one eye remained the most accessible and effective response (Table 1). Unlike auditory, tactile, proprioceptive, or vibratory stimuli—which are harder to control—visual input can be immediately reduced by simply closing one eye. Since vision is estimated to account for up to 80% of the sensory information processed by the brain, even blocking one eye cuts visual input by 50%, offering significant relief [29]. This partial occlusion may have functioned as an instinctive, energy-efficient form of sensory gating, modulating environmental load in real time. In preverbal children with limited behavioral flexibility, such strategies can represent adaptive attempts to downregulate distress before more sophisticated self-regulation skills develop. Notably, as this child matured, he began using more complex compensatory strategies (e.g., requesting sunglasses, avoiding screen time), yet the monocular closure persisted—suggesting it was not simply a reflex or transient tic, but rather a reproducible and intentional behavioral marker of underlying sensory dysregulation.

Within the context of SPD, particularly SOR, such behaviors are interpreted as the child’s intuitive attempts to seek proprioceptive input or deep pressure stimulation, both of which are known to have calming and organizing effects on the central nervous system [30]. Proprioceptive activities, including those providing deep pressure, have been shown to assist in regulating emotional and behavioral responses to sensory stimuli, thereby enhancing the individual’s ability to cope with sensory challenges [13,14]. Deep pressure stimulation can reduce sympathetic arousal, thereby lowering physiological indicators of stress and improving behavioral regulation [31]. These effects are consistent across populations, including children with autism spectrum disorder and sensory integration dysfunction [30], and support the interpretation of such behaviors as self-regulatory rather than purely maladaptive [14]. Thus, these self-initiated behaviors—while atypical in appearance—may reflect neurophysiologically driven attempts to restore sensory homeostasis in the face of environmental overstimulation. The differential diagnosis in this case was challenging, and only in hindsight can we firmly say SPD was the cause. It is important to highlight that our patient does not have ASD: by age 3, the child had typical language, pretend play, and social engagement for his age, making an autism diagnosis highly unlikely. Thus, we consider this a primary SPD case.

### 3.1. Strabismus Reconsidered: A Sensory Processing-Based Framework for Fusion Dysregulation

A direct link between SPD and strabismus has not been systematically investigated in the existing literature. This case challenges the conventional binary classification of ocular alignment as either normal or pathological by introducing a third conceptual axis—sensory modulation.

We propose that strabismus may represent a neurobehavioral manifestation of sensory processing imbalance, rooted within the broader framework of SPD. Specifically, we hypothesize that exotropia and esotropia occupy opposing positions along a continuum of sensory connectivity: exotropia arising from neural underwiring (hyporesponsivity and fusion instability), and esotropia resulting from overwiring (hyperresponsivity and excessive fusion drive).

Our case, positioned within the hyperresponsive domain of SPD, demonstrated frequent monocular eye closure that transiently disrupted fusion. However, this behavior did not result in sustained fusion instability, suggesting that the binocular system in such individuals may be “overwired” to maintain stable single vision despite intermittent occlusion.

**Figure 4:**
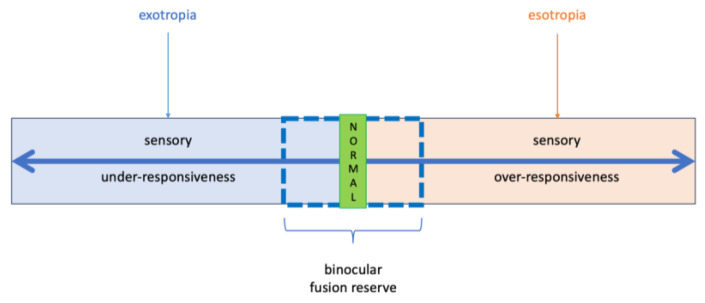
Fusion Dysregulation Hypothesis: esotropia and exotropia reflect opposing poles on a continuum of sensory connectivity.

In early-onset esotropia, this overwiring may become maladaptive, leading to persistent convergence and motor misalignment. Conversely, the hypoconnectivity commonly associated with autism spectrum disorder may predispose individuals to intermittent or constant exotropia due to compromised fusion maintenance. Notably, clinical studies of ASD have reported a higher prevalence of exotropia relative to esotropia [32], consistent with the idea that conditions involving reduced neural connectivity and sensory under-responsivity might favor divergent strabismus. Our proposed *Fusion Dysregulation Hypothesis* reframes strabismus within a sensory integration framework (Figure 4).

Specifically, we introduce a functional continuum of sensory modulation from under-responsivity (hypoconnectivity) to hyperresponsivity (overwiring), aligned with the neural signatures of SPD and ASD. We posit that even in the absence of structural brain anomalies, dysregulated sensory processing alone can yield similar phenotypes through disrupted fusion dynamics. This divergence highlights the importance of considering sensory profiling in children with atypical ocular behaviors, irrespective of overt neurologic pathology. Thus, our model integrates neurodevelopmental and sensory domains into a broader, multidimensional understanding of strabismus.

Our hypothesis underscores the need for a more integrated clinical approach. We advocate for structured sensory profiling in the assessment of strabismic children, and reciprocally, for ophthalmologic evaluation in children diagnosed with SPD. This bidirectional strategy may uncover atypical visual behaviors, enhance diagnostic precision, and foster interdisciplinary collaboration to address underlying neurodevelopmental dynamics more effectively.

### 3.2. Limitations

This report describes a single case, which inherently limits generalizability. We cannot definitively conclude causality from an N = 1 observation; the *Fusion Dysregulation Hypothesis* remains a conceptual framework requiring further empirical validation.

While retrospective interpretation may raise concerns about observer bias, it is important to clarify that the conclusion of SPD was reached only after the exclusion of ophthalmologic, neurologic, and synkinetic diagnoses, rather than through any *a priori* hypothesis.

Furthermore, objective assessments of sensory reactivity (e.g., standardized sensory profiles) and fusion stability (e.g., eye-tracking during stimulus presentation) were not feasible in this case. This limitation stems from the child’s developmental stage and limited cooperation during clinical evaluations. Additionally, the child’s symptoms primarily occurred in naturalistic settings—specifically outdoors, in response to sunlight, wind, and environmental motion—which could not be reliably replicated under standard examination conditions. Notably, the child tolerated torchlight in the clinic without distress, underscoring the context-specific nature of his sensory responses.

The child had only recently been referred to occupational therapy at the time of manuscript submission, and as such, no therapeutic outcomes were available for evaluation. We chose not to include preliminary or anecdotal impressions, as they would not meaningfully advance the broader proof-of-concept hypothesis, which requires validation through future empirical studies.

Finally, elements of the proposed mechanism—such as “overwiring” of fusion pathways—are extrapolated from neurodevelopmental and sensory models, and remain speculative in the absence of direct neurophysiological evidence.

### 3.3. Future Direction

To explore the proposed sensory–strabismus link, case–control studies should examine whether specific sensory processing traits correlate with strabismus subtypes. In parallel, children with SPD should be evaluated for subtle binocular anomalies, even when overt strabismus is absent. Neuroimaging techniques (e.g., diffusion MRI, rs-fMRI) may help uncover sensory–motor integration differences in affected children. Ultimately, research should assess whether sensory integration therapies can serve as effective adjuncts to conventional strabismus treatment in select cases.

## 4. Conclusions

This case supports a novel theoretical framework situating strabismus along a continuum of sensory processing regulation. By integrating concepts from SPD and ASD into the understanding of binocular vision anomalies, we propose that exotropia and esotropia may reflect opposing sensory states rooted in neural connectivity. The patient’s stable fusion despite frequent eye closure supports the notion of overwiring within the sensory-fusion axis. As a proof-of-concept, this single case suggests that sensory processing factors may contribute to pediatric strabismus presentations; however, larger, controlled studies are needed to validate this proposed association. Traditional structural or muscular etiologies do not adequately explain all strabismic presentations; therefore, we advocate for systematic sensory evaluations in children with ocular misalignment. Likewise, visual examinations should be integrated into the assessment of SPD, as atypical ocular behaviors may signal broader sensory dysregulation. This integrative model invites a paradigm shift, encouraging collaboration across ophthalmology, occupational therapy, and developmental neuroscience to enhance diagnostic precision and therapeutic strategies for affected children.

## Figures and Tables

**Table 1:** Developmental progression of sensory triggers and the child’s responses. Behavioral responses became more complex in parallel with the emergence of age-appropriate motor and cognitive abilities.

Age	Sensory Triggers	Observed Responses	Developmental Context
2 months	Breastfeeding Camera flash	Tight right-eye closure; Facial grimacing	Reflexive avoidance of multisensory input due to limited motor control; Grimacing as a primitive motor strategy to reinforce eye closure in the absence of voluntary self-regulation
3–4 months	Camera flash	Eye poking	Emergence of hand coordination; Early attempt at self-directed monocular eye closure
8–10 months	Bright sunlight Camera flash Strong breeze Air puff Spoon feeding	Frequent monocular/binocular eye blinking	Growing visual awareness; Beginning intentional avoidance responses; Voluntary control required for monocular winking is not yet present
1–2 years	Moderate light or wind, especially during teething pain	Prolonged monocular closure (sometimes alternating); Eye/face rubbing	Improved fine motor skills allow for tactile self-regulation; Lower sensory threshold noted during teething
	Falling snowflakes	Eye closure Head turning away Covering face with hands Mental distress	Increased sensory-motor integration; Distress triggered by combined visual, tactile and vibratory stimuli
2–3 years	Vibrations from digger	Eye closure	Increased sensory-motor integration; Distress triggered by combined visual, tactile and vibratory stimuli, attempts to physically reject stimulus
	Bright light, wind, digital screens	Requests sunglasses; covers eye with hand/fist; Limits screen time; Rubs eyes/forehead; Pulls hair; face pinching shields face in wind	Developed cognitive awareness of triggers; Initiates preventive and self-regulatory behaviors (e.g., requesting sunglasses, discontinuing screen use); Combines visual avoidance with proprioceptive strategies

## Data Availability

The original contributions presented in this study are included in the article. Further inquiries can be directed to the corresponding author.
